# The 2014 Ebola virus outbreak in West Africa highlights no evidence of rapid evolution or adaptation to humans

**DOI:** 10.1038/srep35822

**Published:** 2016-10-21

**Authors:** Xingguang Li, Junjie Zai, Haizhou Liu, Yi Feng, Fan Li, Jing Wei, Sen Zou, Zhiming Yuan, Yiming Shao

**Affiliations:** 1 State Key Laboratory for Infectious Disease Prevention and Control, National Center for AIDS/STD Control and Prevention, Chinese Center for Disease Control and Prevention, Beijing, China; 2 Collaborative Innovation Center for Diagnosis and Treatment of Infectious Diseases, Hangzhou, Zhejiang, China; 3 Key Laboratory of Agricultural and Environmental Microbiology, Wuhan Institute of Virology, University of Chinese Academy of Sciences, Wuhan, Hubei, China; 4 Centre for Emerging Infectious Diseases, State Key Laboratory of Virology, Wuhan Institute of Virology, University of Chinese Academy of Sciences, Wuhan, Hubei, China

## Abstract

Following its immergence in December 2013, the recent Zaire Ebola virus (EBOV) outbreak in West Africa has spread and persisted for more than two years, making it the largest EBOV epidemic in both scale and geographical region to date. In this study, a total of 726 glycoprotein (GP) gene sequences of the EBOV full-length genome obtained from West Africa from the 2014 outbreak, combined with 30 from earlier outbreaks between 1976 and 2008 were used to investigate the genetic divergence, evolutionary history, population dynamics, and selection pressure of EBOV among distinct epidemic waves. Results from our dataset showed that no non-synonymous substitutions occurred on the GP gene coding sequences of EBOV that were likely to have affected protein structure or function in any way. Furthermore, the significantly different *dN*/*dS* ratios observed between the 2014 West African outbreak and earlier outbreaks were more likely due to the confounding presence of segregating polymorphisms. Our results highlight no robust evidence that the 2014 EBOV outbreak is fast-evolving and adapting to humans. Therefore, the unprecedented nature of the 2014 EBOV outbreak might be more likely related to non-virological elements, such as environmental and sociological factors.

The Zaire Ebola virus (EBOV), a member of the *Filoviridae* family, is an enveloped, non-segmented, negative strand RNA virus approximately 19 kb in length, which has caused considerable morbidity and mortality in West African human populations^1^. EBOV is the causative agent of Ebola virus disease (EVD), which was first isolated and identified in 1976 during a hemorrhagic fever epidemic in the Democratic Republic of Congo (DRC) (then known as Zaire, with the outbreak epicenter located in Yambuku), with cases reported in several districts of Guinea, suggesting a hidden, at that point, history of pandemic spread in Africa. The most recent outbreak, beginning in the forested areas of south eastern Guinea in West Africa in December 2013, is unprecedented in its magnitude, geographic occurrence, severity, persistence, and complexity, and has spread to the neighboring countries of Sierra Leone, Liberia, Nigeria, Senegal, and Mali, as well as Europe (Italy, Spain, and United Kingdom) and North America (United States of America)^2^,^3^,^4^. As of 27 March, 2016, the World Health Organization (WHO) has reported 28646 cases of EVD and 11323 deaths worldwide^5^. Notably, the significant characteristic of this epidemic is that it has spread into major urban centers in West Africa, further facilitating its continued spread from human to human^4^. In an ongoing public health crisis, where accurate and real-time digital pathogen surveillance is crucial, phylogenetic and selection analyses have become ever more powerful and rapid in extracting molecular epidemiological information from sequence data to understand pathogenic origin, transmission dynamics, evolution, and adaptation to host populations^6^,^7^,^8^,^9^,^10^.

Understanding the natural selection that has shaped genetic variation is a critical aspect for the evaluation of how certain pathogens adapt to host populations. Great efforts have been made in recent decades to understand and identify the “molecular footprint” left in nucleotide and protein-coding sequences by the past action of natural selection. Therefore, identification of genes or genomic regions that have been targeted by natural selection, then pinpointing the genotypic variation that causes a certain pathogen to adapt to a host population, is essential for the development of vaccines and therapeutics. Comparison of the relative rates of non-synonymous (*dN*) and synonymous substitutions (*dS*) can provide information on the type of selection that has acted on a given set of protein-coding sequences, and has become a standard measure of selective pressure^11^. The *dN/dS* ratio is also referred to as the ω ratio or *K*_*A*_/*K*_*S*_, with ~1, <1, and >1 indicating neutral evolution, negative or purifying selection, and positive or diversifying selection, respectively.

In the present study, we employed state-of-the-art methods to investigate the genetic divergence and evolutionary dynamics and selection of EBOV between distinct outbreaks based on 756 GP gene sequences of EBOV with known sampling dates and geographic locations. Our study could provide insights into the origin and evolutionary history of EBOV epidemic waves in West Africa.

## Materials and Methods

### Sequence dataset

All available complete genome GP gene sequences (as of 1 September, 2015) of EBOV from the period of 1976–2015 with known sampling dates and geographic locations were retrieved from the GenBank database of the National Center for Biotechnology Information (http://www.ncbi.nlm.nih.gov/). In cases of multiple sequences per patient, only one sequence was randomly selected. The final dataset included 756 GP gene sequences (Supplementary Table 1). The sampling locations were Sierra Leone (n = 466), Guinea (n = 235), DRC (n = 23), Liberia (n = 21), Gabon (n = 7), and Mali (n = 4). The dataset was used to estimate the evolutionary rate, perform time-scaled phylogenetic analyses, and assess selection. All sequences were aligned using MAFFT v7.222^12^ and then adjusted manually using BioEdit v7.2.5^13^. Multiple sequence alignments were screened for recombinant viral sequences using RDP v4.36^14^ and no recombinant sequences were identified. Finally, jModelTest 2 was used to select the substitution model that adequately fit the sequence dataset^15^.

### Likelihood-mapping analysis and evolutionary divergence estimates

To study the amount of evolutionary information contained in the dataset, likelihood-mapping analysis^16^, based on the maximum-likelihood values for the three possible unrooted trees of four randomly chosen sequences, was performed using TREE-PUZZLE v5.3^17^, in which all possible quartets for each of the six lineages representing specific epidemics were analyzed. The analysis was then repeated for the entire genealogy. For each quartet, the likelihood-mapping method partitions the area of an equilateral triangle into seven regions. The three trapezoids at the corners represent the areas supporting strictly bifurcating trees. The three rectangles on the sides represent regions where the decision between two trees is not obvious. The center of the triangle represents sets of points where all three trees are equally supported. The three likelihoods for the three tree topologies of each possible quartet (or of a random sample of quartets) are reported as a dot in an equilateral triangle. The distribution of points in the seven areas of the triangle gives an impression of the tree-likeness of the data. Thus, the three corners represent fully resolved tree topologies; the center represents star-like phylogenies; and the three areas on the sides represent network-like phylogeny, where the data support conflicting tree topologies. Intra and inter-lineage evolutionary divergence estimates for each of the six distinct lineages were calculated using a maximum composite likelihood model^18^ with gamma distribution in MEGA v5.05^19^.

### Phylogenetic tree construction

Maximum-likelihood (ML) phylogenetic trees were constructed using RAxML v8.0.9^20^ with the GTRCAT nucleotide substitution model and GARLI v2.1 web service^21^. The reliability of the tree topology or branching order was evaluated by bootstrap analysis with 1000 replicates. The final ML trees were visualized using FigTree v1.4.2 (http://tree.bio.ed.ac.uk/software/figtree).

### Molecular clock analysis and evolutionary rate estimates

To investigate the temporal signal and “clocklikeness” of molecular phylogenies of the dataset, linear regression was performed on the root-to-tip distances of samples versus the date of the isolate on a ML phylogeny inferred using RAxML v8.0.9^20^ via the program Path-O-Gen v1.4 (http://tree.bio.ed.ac.uk/software/pathogen/) and second by checking the posterior distribution of the coefficient of variation statistics. Evolutionary rates were estimated using Bayesian Markov Chain Monte Carlo (MCMC)^22^ in BEAST v1.8.2^23^. The BEAGLE parallel computation library was used to enhance the speed of the likelihood calculations^24^,^25^. The alignment was partitioned into first + second codon positions and third codon positions (giving a total of two partitions). The nucleotide substitution process was modeled independently for each partition with HKY + γ4^26^,^27^. A Skygrid non-parametric coalescent model^28^ and strict molecular clock were selected. An uninformative continuous time Markov chain (CTMC) reference prior was used on the rate of evolution^29^. Bayesian MCMC chains were run for 500 million generations, 10% of which were removed as burn-in, and sampled every 50000 steps. Convergence and uncertainty in parameter estimates were evaluated by calculating effective sample size (ESS) and 95% high probability density (HPD) values, respectively, in Tracer v1.6 (http://beast.bio.ed.ac.uk/software/tracer). Maximum-clade credibility (MCC) trees summarizing all MCMC samples were generated using TreeAnnotator v1.8.2 with a burn-in rate of 10%^23^.

### Estimating global rates

Comparing the global estimates of synonymous (*dS*) and non-synonymous (*dN*) substitutions averaged over the entire alignment can provide a crude measure of the overall strength of selection on the coding region. Global estimates of *dS* and *dN* can be obtained by fitting a codon substitution model to a given alignment and corresponding tree. Global *dN*/*dS* ratio estimation was performed using the AnalyzeCodonData.bf batch file available under the Standard Analyses menu in HyPhy v2.2.4 (http://www.hyphy.org)^30^. HyPhy is an open-source software package used for the analysis of genetic sequences using techniques in phylogenetics, molecular evolution, and machine learning.

### Interclade variation in substitution rate

To evaluate the hypothesis of whether the *dN*/*dS* ratio varied significantly between the 2014 West African outbreak lineage and earlier outbreak lineages, the internal branch that separated clades of interest was specified using the SelectionLRT.bf batch file, executed through the Standard Analyses menu under the Compartmentalization submenu in HyPhy v2.2.4^30^. To determine whether a nested model provides a significant improvement of fit to the dataset, it reports *p*-values from likelihood ratio tests and Akaike information criterion values, which adjust likelihood ratios for differences in the number of model parameters. It also provides 95% confidence bounds, derived from the likelihood profile, for estimates of the *dN*/*dS* ratio for each clade and the internal branch, respectively.

### Comparing internal and terminal branches

A useful hypothesis that can be tested in viral sequences is whether the relative rate of non-synonymous substitution varies between terminal and internal branches of a tree. This hypothesis can be evaluated by the TestBranchDNDS.bf batch file in HyPhy v2.2.4, which is found in the Standard Analyses menu, under the heading Positive Selection^30^. This batch file attempts to fit a global model in which non-synonymous substitution is constrained to the same value (SharedNS1) on all branches, before relaxing this constraint for an arbitrary selection of branches.

### Evolutionary interactions between sites

A Bayesian Graphical Model (BGM) was used to reconstruct evolutionary histories of individual sites to find evidence of co-evolution between sites in our dataset by the QuickSelectionDetection.bf batch file in HyPhy v2.2.4, which is found in the Standard Analyses menu, under the heading Positive Selection^30^. The BGM holds two main advantages over pairwise association tests: firstly, it can distinguish between direct and indirect associations between variables; and secondly, it can provide an efficient and compact representation of joint probability distribution in an accessible graphic format^31^. This “evolutionary-network” model was applied to detect interactions among codon sites in the GP gene sequences of EBOV. In total, 12 BGM analyses were processed under various combinations of three parameters: including sites with a set number of total substitutions (value could be 1, 2, or 3, default 1), maximum parents per node (1 or 2, default 1), and ancestral resampling (no or yes). The other parameters for the BGM analyses were set to default values.

### Selection analysis

To assess non-synonymous/synonymous substitution rates (i.e., the *dN/dS* ratio, ω ratio, or *K*_*A*_/*K*_*S*_) for all GP codon sites and all branches of the phylogeny of EBOV, three different model classes were examined using the CODEML program in phylogenetic analysis by maximum likelihood (PAML) v4.8^32^ on the basis of GP codon sequence alignments of EBOV: (i) branch-specific models, (ii) site-specific models, and (iii) branch-site models.

Branch-specific models permit heterogeneity in the ω ratio among branches in the phylogeny previously defined as foreground branches^33^. They are specified using the variable model. One-ratio (null hypothesis) and two-ratio (alternative hypothesis) model comparison can be used to test whether the ratio for the foreground clade is significantly different from that of the background clade. The likelihood ratio test has d.f. = 1.

For site-specific models, four pairwise codon-based substitution models are compared. Briefly, the M0 model, which allows one ω ratio for all branches, is calculated as the simplest null model and compared with the more sophisticated alternative discrete model M3, which allows the ω ratio to vary among sites, but holds ω constant among branches for consistency^32^,^34^. Furthermore, M1a (nearly neutral) allows for two rates of ω to vary between 0 and 1, while M2a (positive selection) allows for an additional rate of ω > 1. Similarly, M7 (neutral) estimates ω with a beta-distribution over the internal branches (0, 1), whereas M8 (selection) adds parameters to M7 for an additional class of codons with a freely estimated ω value. However, M8a (neutral) is a special case that fixes the additional codon class at a ω value of one. The two pairwise site-specific models of M1a-M2a and M7-M8 are particularly useful, forming two likelihood ratio tests of positive selection. In both tests d.f. = 2 should be used. In addition, M1a and M2a are slight modifications of M1 (neutral) and M2 (selection), respectively. The M1a-M2a comparison appears to be more robust (but less powerful) than the M7-M8 comparison. The single likelihood ancestor counting (SLAC) method^35^ for evaluating site-specific levels of selection using the QuickSelectionDetection.bf batch file in HyPhy v2.2.4, which is found in the Standard Analyses menu under the heading Positive Selection^30^, was used for confirming the results of site-specific models implemented in the CODEML program of PAML v4.8^32^.

Branch-site models allow ω to vary in foreground branches, but also feature heterogeneity in selective pressures throughout the sequences by defining different codon site-classes with different ω ratios^36^. Comparison between branch-site model A, with ω fixed at one for the examined branches as the null model, and model A, featuring an extra class of sites under positive selection with ω > 1 in the foreground branches, was used as a conservative test to detect positive selection as opposed to relaxed purifying selection affecting a few sites in the selected branch. In likelihood ratio tests, d.f. = 1. Branch-site model A, as the alternative hypothesis, can be compared with the new site model M1a (nearly neutral), as the null hypothesis, with d.f. = 2, which is also known as branch-site test 1. This test can be significant when the foreground branches are either under relaxed selective constraint or positive selection^37^,^38^. Finally, the significant likelihood ratio test comparing branch-site model A1 (alternative hypothesis) to M1a (null hypothesis) indicates relaxed constraint^36^. To measure divergent selection pressures acting on a significant number of codon sites, a test comparing branch-site model D (alternative hypothesis), which accommodates both heterogeneity among sites and divergent selective pressures, and site-specific null discrete model 3 (null hypothesis), which assumes variation among sites but no variation among branches, thus allowing a class of sites to be under divergent selection pressure between foreground branches and the rest of the tree, was used^39^. In this study, the assumed topology was an unrooted ML tree, therefore, the likelihood ratio test used d.f. = 1.

Likelihood ratio tests were used to test for the statistical significance of the signal of selection^40^ in various models implemented in PAML v4.8^32^. The statistical significance of the likelihood ratio test was calculated assuming that twice the difference in the log of maximum likelihood between two nested models exhibited chi-squared distribution, with the degrees of freedom given by the difference in the number of parameters in the two models^36^,^41^. When the likelihood ratio tests were significant, the Bayes empirical Bayes approach^37^ was used to identify codon sites likely to have potentially evolved under positive selection, based on a posterior probability threshold of 0.95, from site-specific models M2a and M8 and branch-site model A. In addition, the 2014 West African outbreak lineage and earlier outbreak lineages were designated as foreground and background branches, respectively.

## Results

### Phylogenetic analyses

Phylogenetic analyses of both RAxML and GARLI trees confirmed the phylogenetic position shown in recent study^4^ and divided the EBOV epidemics into six lineages based on the temporality of each epidemic wave and phylogenetic clustering (Fig. S1). The six lineages are as follows: 1976–1977 epidemic in DRC (lineage I); 1995 epidemic in DRC (lineage II); 1994/1996 epidemics in Gabon (lineage III); 2002–2003 epidemic in DRC and Gabon (lineage IV); 2007–2008 epidemic in DRC (lineage V) and 2014–2015 epidemic in Sierra Leone, Guinea, Liberia, and Mali (lineage VI). Our phylogenetic analyses also exhibited staircase-like topology, in accordance with previous studies^4^,^9^,^10^,^42^,^43^,^44^,^45^. The branch lengths of EBOV lineages tended to show correlation with time of recorded outbreaks, except for lineage V, which was much shorter than lineage IV, also in accordance with previous studies^4^,^9^,^10^,^43^. Furthermore, the genealogies also demonstrated the emergence of two clades (composed of lineages II-III and lineages IV-VI, respectively) after the 1976–1977 epidemic in DRC, although the phylogenetic positions of lineages IV and V in the two constructed ML phylogenetic trees were not consistent with each other.

### Likelihood-mapping and evolutionary divergence analyses

The amount of evolutionary information for each distinct epidemic and the epidemics overall were further investigated by likelihood-mapping analysis^16^,^17^. The evaluation of each possible quartet (group of four sequences) for each lineage of sequences sampled during distinct EBOV epidemic waves demonstrated significant differences in phylogenetic signal and the amount of evolutionary information between epidemics in the dataset (Fig. S2). Given that there were only three sequences, lineage IV was excluded from this analysis. The most notable result of the likelihood-mapping analysis revealed that 100% of the quartets from lineage V were distributed in the center of the triangle, indicating a strong star-like phylogenetic signal reflecting a new lineage, which might be due to exponential epidemic spread. Intriguingly, lineage V was characterized by the lowest genetic divergence among the six distinct EBOV epidemics (Fig. S3), which further confirmed that lineage V was most likely a new lineage, in accordance with likelihood-mapping analysis^16^,^17^. Likewise, 83.3% and 85.8% of the quartets from lineages II and VI, respectively, were distributed in the center of the triangles and showed lower genetic divergence. Notably, 59% of the quartets from lineage III were distributed in the center of the triangle, but with higher genetic divergence. In contrast, 39.9% of the quartets from lineage I were distributed in the center of the triangle, but with lower genetic divergence. Also of note, 83.7% of the quartets from the overall epidemics were distributed in the center of the triangle, but with higher genetic divergence (data not shown). We also observed that lineage IV had the highest genetic divergence among the six distinct EBOV epidemics.

### Demographic analysis

**Figure 1:**
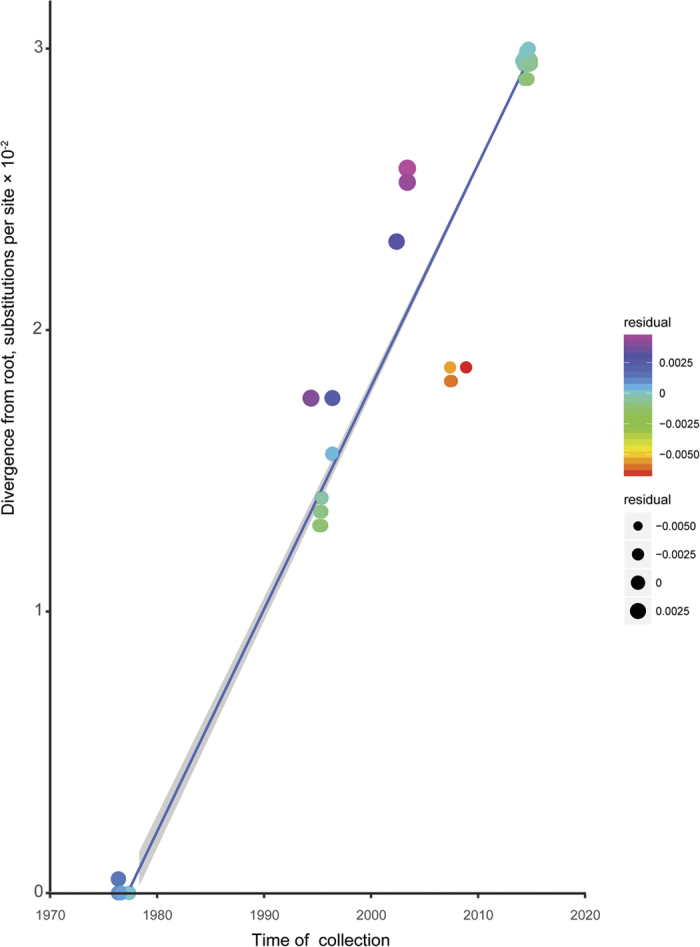
Root-to-tip regression of a maximum likelihood (ML) phylogenetic tree of GP gene sequences of EBOV. The residual of each GP gene sequence of EBOV is highlighted using different colors and circles.

**Figure 2:**
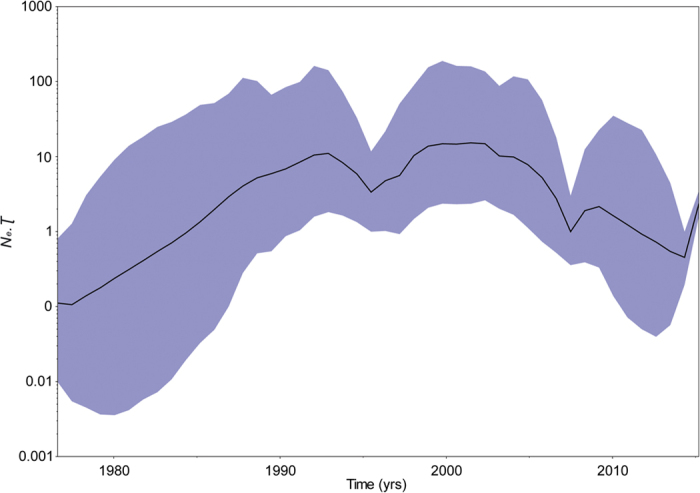
Estimation of past population dynamics of EBOV isolated from West Africa using the Bayesian coalescent-based non-parametric skygrid model. Left-hand axis represents effective number of infections (*N*_*e*_) multiplied by mean viral generation time (τ). Black line and shaded blue region represent the median and 95% high posterior density (HPD) intervals of EBOV past population dynamics, respectively.

**Figure 3:**
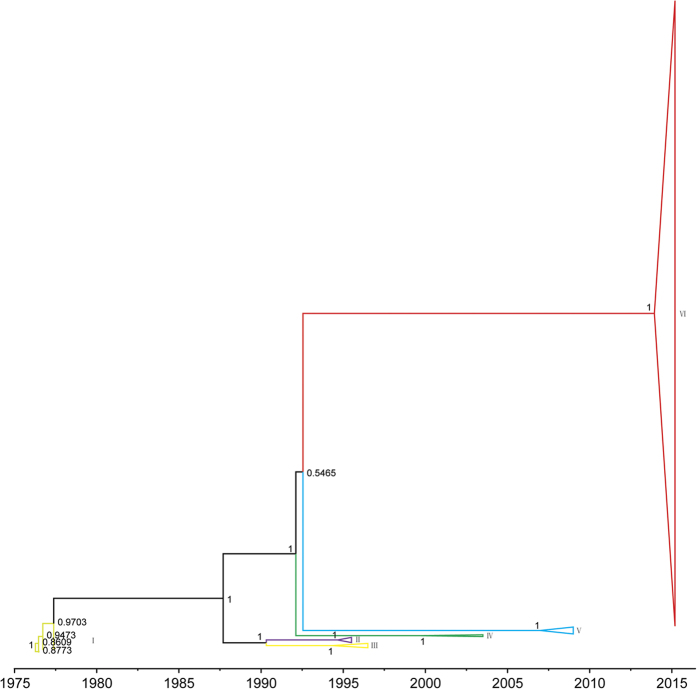
Bayesian maximum clade credibility (MCC) phylogenetic tree of GP gene sequences of EBOV isolated from West Africa. Branch lengths are scaled in time by enforcing a strict molecular clock. Posterior probability for major nodes is shown.

The relationship between genetic divergence and time using least squares regression showed that sequences from lineage I were very close to the root of genealogy (Fig. 1). The Bayesian time-scaled phylogenetic analysis estimated an average rate of evolution over the EBOV GP gene sequences of about 1.025e-3 substitutions per site per year (95% HPD interval: 8.17e-4–1.217e-3) and dated the time of the most recent common ancestor (TMRCA) of the sampled viruses as 1976.3 (95% HPD interval: 1975.9–1976.5), in accordance with earlier estimates^9^,^43^ and known epidemiological data^46^. We further investigated the population dynamics of the 1976–2015 EBOV by estimating a coalescent-based Bayesian skygrid plot for the dataset^28^, which depicted the changes in effective population size over time. As illustrated in Fig. 2, the effective population size of the 1976–2015 EBOV experienced complex dynamics characterized by four different rapid expansion phases interleaved by three different declining growth periods. Intriguingly, the effective population size of lineage VI experienced a steep rapid expansion, in agreement with known epidemiological data^3^,^47^. In addition, the topology of the Bayesian MCC tree of the GP gene sequences of EBOV was consistent with the ML phylogenetic trees constructed by RAxML v8.0.9^20^ and GARLI v2.1^21^ (Fig. 3).

### Global rates and branch-by-branch variation

The global *dN*/*dS* ratio estimate using the AnalyzeCodonData.bf batch file in HyPhy v2.2.4^30^ was R = 1.127, indicating nearly neutral evolution. Comparing selective pressures between the 2014 West African outbreak and earlier outbreaks using the SelectionLRT.bf batch file in HyPhy v2.2.4^30^ resulted in global *dN*/*dS* ratios of 0.600 (95% confidence bounds: 0.461–0.763) and 0.342 (95% confidence bounds: 0.254–0.449), respectively. The estimation for the internal branch separating the 2014 West African outbreak and earlier outbreaks was 0.194 (95% confidence bounds: 0.101–0.334), indicating a lower value compared with the above two branches. The likelihood ratio test comparing the single rate model resulted in a *p*-value of 0.008, indicating significantly different global *dN*/*dS* ratios between the 2014 West African lineage and earlier lineages. However, testing global *dN*/*dS* variation among branches using the TestBranchDNDS.bf batch file in HyPhy v2.2.4^30^ showed that the relative rate of non-synonymous substitution variation between terminal and internal branches was not significant (*p* = 0.249) and SharedNS1 was ~0.305. Most notably, the 12 BGM analyses showed zero edges with high (≥0.95) posterior support, although a small number of edges with weak (≥0.5) posterior support were identified.

### Selection analysis

The one-ratio model (M0) yielded an estimated ω of 0.379, indicating that purifying selection dominated the evolution of the EBOV GP gene. This finding was based on an average over all sites and branches. However, the one and two-ratio model comparisons of branch-specific models using the CODEML program in PAML v4.8^32^, which provided a likelihood score for each model, subsequently used to test for significance between the null and alternative hypotheses, showed significantly different *dN*/*dS* ratios between the 2014 West African outbreak and earlier outbreaks (*p* = 0.023, Supplementary Table 2), in accordance with the SelectionLRT.bf batch file analysis implemented in HyPhy v2.2.4^30^. To investigate whether different environmental factors were driving the emergence of new EBOV variants in specific outbreaks, site-specific models were examined using CODEML^32^ to identify the presence of selective pressures on specific codon sites of the EBOV GP gene (Supplementary Table 2). Likelihood ratio tests of M0 against M3 indicated significant variation in selective pressures among sites. However, comparisons of the two pairwise site-specific models of M1a and M2a, and M7 and M8 showed that there were no codon sites identified as being under positive selection. Intriguingly, the *p*-value rejected M8a when compared with M8. The BEB method identified a small number of codon sites under positive selection, but none reached a posterior probability of 95%^37^. Selection was further investigated using SLAC in HyPhy v2.2.4^30^. The results indicated that no codon sites were under positive selection (*p* ≤ 0.1), in accordance with the PAML analyses^32^. The evolutionary rates of change at the codon level also supported the PAML analysis results^32^. The evolutionary rate for the third codon position (1.519 [95% HPD interval: 1.33–1.72]) was significantly greater than that of the first + second codon position (0.741 [95% HPD interval: 0.642–0.833]), which might indicate no or extremely low levels of positive selection. The results of branch-site models for positive selection also showed that no statistically significant codon sites were identified in the dataset (Supplementary Table 2). Simultaneously, no relaxed selective constraint or positive selection were detected for the 2014 West African outbreak compared with earlier outbreaks (Supplementary Table 2). However, comparison of branch-site Model D and M3 strongly indicated divergent selection pressures at the sequence level (*p* = 0.014). Parameter estimates under Model D with *k* = 3 site classes indicated a large set of sites (56.338%) evolving under strong purifying selection (ω = 0.01242), a small set of sites (1.13%) evolving under selective pressure (ω = 6.28165), and a large set of sites (42.532%) evolving under divergent selective pressure, with purifying selection in earlier outbreaks (ω = 0.29709) and weak purifying selection in the 2014 West African outbreak (ω = 0.86160).

## Discussion

On 29 March, 2016, the WHO stated that the EBOV situation in West Africa no longer constituted a public health emergency of international concern and the temporary recommendations adopted in response to the outbreak could be terminated^48^. It should be noted that the recent EBOV epidemic continued for over two years, the longest EBOV outbreak to date, causing a devastating impact to West African populations. In addition, unlike previous EBOV epidemics, which caused zoonotic outbreaks in human populations and remained localized, the recent EBOV epidemic showed sustained transmission among human populations and spread across many West African countries. These findings give the impression that EBOV is fast-evolving and possibly adapting to humans. Although our understanding of the establishment and evolution of human pathogens has increased, we know remarkably little about the early dissemination routes of EBOV or its detailed cross-species transmission history from nonhuman primate hosts to humans. The lack of direct evidence regarding the early transmission of the recent EBOV epidemic has led to several competing hypotheses on its outbreak. The two most widely accepted hypotheses argue that non-virological factors, such as urbanization, and/or viral genetic factors, such as virus infectivity, virus antigenic variation, and human immune system adaptation, are responsible for the virulence and duration of the most recent outbreak.

The GP gene is the most widely studied of the EBOV genome, and its expression on the particle surface is the only transmembrane protein of EBOV responsible for receptor binding and membrane fusion^49^,^50^. As such, it is an essential component of pathogenicity as well as the primary target of antibodies^51^,^52^,^53^. However, by probing information contained in sampled viral sequences, few studies have comprehensively assessed the dynamic nature, molecular change, and selection of the GP gene in EBOV. In the present study, phylogenetic analyses identified six main distinct lineages of EBOV, approximately corresponding to six main outbreaks, with strong nodal support (Fig. 3 and Fig. S1). Viral sequences from the same period or location were most likely to form a distinct lineage, which indicates that the EBOV population may have experienced bottleneck or founder effects. Furthermore, the GP gene sequences could be divided into two clades after the 1976–1977 epidemic in DRC. One clade was composed of lineages II-III from two countries (DRC and Gabon), while the other clade consisted of lineages IV-VI from six countries (DRC, Gabon, Sierra Leone, Guinea, Liberia, and Mali). Notably, likelihood-mapping analysis^16^,^17^ showed that 83.7% of the quartets from lineages I-VI were distributed in the center of the triangle. This highlights the difficulty in obtaining a high resolution phylogenetic tree using the GP gene sequences of all EBOV outbreaks from the dataset, which resulted in star-like topology, especially for lineage V (Fig. S2). Therefore, it might be possible to obtain better topology with a higher degree of resolution for the GP gene sequences by excluding lineage V from the present study. As such, it is tempting to speculate that the putative EBOV reservoirs for lineage V might be distinctly different from that of other lineages. However, so few EBOV samples have been isolated from nonhuman hosts that we have little idea about the evolutionary and epidemiological patterns of the six different lineages of EBOV from the infected human populations within their origin reservoirs. Identification and characterization of the animal reservoirs for EBOV might emerge to support such conclusions. Our estimation of the evolutionary rate of the GP gene was about 1.025e-3 substitutions per site per year (95% HPD interval: 0.817e-3–1.217e-3) and TMRCA was 1976.3 (95% HPD interval: 1975.9–1976.5), consistent with previous studies^9^,^43^. Therefore, no evidence of a change in evolutionary rate over the course of the current epidemic was observed. It should be noted, however, that knowing the evolutionary rate alone provides almost no information regarding the likelihood that a virus will adapt to a host population. Notably, coalescent-based skygrid plot^28^ reconstruction demonstrated a complex dynamic population for EBOV (Fig. 2).

Both PAML v4.8^32^ and HyPhy v2.2.4^30^ were used to investigate selection pressures acting on GP gene sequences of EBOV. Significantly different *dN*/*dS* ratios between the 2014 West African outbreak and earlier outbreaks were detected, suggesting significantly positive selection or purifying selection (according to ω values), or just strictly or relaxed selective constraints or divergent selection pressures in the 2014 West African outbreak. However, no evidence of positive selection was detected (Supplementary Table 2), which is corroborated by earlier research^54^. In addition, no relaxed selective constraints or co-evolution codon sites were detected by branch-site models^32^ or BGM approaches^30^, respectively. Intriguingly, significantly divergent selection pressures acting on the coding sequence of the GP gene between the 2014 West African outbreak and earlier outbreaks, and strong purifying selection acting on a large set of sites (56.338%) of the GP coding sequence, were detected by branch-site models^32^. It is tempting, therefore, to speculate that no non-synonymous substitutions occurred on the GP gene coding sequences of EBOV that were likely to affect protein structure or function in any way. Therefore, the null hypothesis of neutral evolution for GP gene sequences of EBOV cannot be rejected based on our selection analyses, and the significantly different *dN*/*dS* ratios between the 2014 West African outbreak and earlier outbreaks might be more likely due to the confounding presence of segregating polymorphisms. Therefore, greater attention should be paid to accurately estimate the evolutionary rate under the molecular-clock model when many segregating polymorphisms exist, rather than fixed mutations from a population sampled over a short time scale. The lack of positive pressure differences and the rate of evolution distinction between the 2014 West African outbreak and earlier outbreaks offers no convincing evidence that rapidly evolving or non-synonymous mutations occurred in the 2014 West African outbreak. Until more genomic sequence data from this outbreak are obtained, it is not possible to say more about the likelihood that this lineage will adapt to human populations. As such, non-virological factors such as human population growth, progressive urbanization, and poor health facilities might more likely explain the magnitude of the 2014 West African outbreak^55^,^56^. Taken together, continued genomic surveillance of the epidemic, especially from animal reservoirs sampled during the outbreak and across the geographical range, combined with epidemiological investigation and clinical recognition, are required to trace the history of recent epidemic waves. This is increasingly important in terms of EBOV intervention strategies, and will further guide research on vaccines and therapeutic targets.

## Additional Information

**How to cite this article**: Li, X. *et al.* The 2014 Ebola virus outbreak in West Africa highlights no evidence of rapid evolution or adaptation to humans. *Sci. Rep.* **6**, 35822; doi: 10.1038/srep35822 (2016).

## Supplementary Material

Supplementary Information
