# Supplementary material for: The 2014 Ebola virus outbreak in West Africa highlights no evidence of rapid evolution or adaptation to humans
Source: Sci Rep. 2016 Oct 21;6:35822. doi: 10.1038/srep35822 (PMC5073338; doi:10.1038/srep35822)
Supplement: Supplementary Information [file srep35822-s1.pdf]

The current 2014 Ebola virus outbreak in West Africa highlights no evidence of rapid evolution or adaptation to humans

Xingguang LI[1, #], Junjie ZAI[2, #], Haizhou LIU[3, #], Yi FENG[1], Fan LI[1], Jing WEI[1], Sen ZOU[1], Zhiming YUAN[2, *] and Yiming SHAO[1, *]

[#]These authors contributed equally to this work. [*]These authors contributed equally to this work.

1. State Key Laboratory for Infectious Disease Prevention and Control, National Center for AIDS/STD Control and Prevention, Chinese Center for Disease Control and Prevention, Beijing, China. Collaborative Innovation Center for Diagnosis and Treatment of Infectious Diseases, Hangzhou, Zhejiang, China.
2. Key Laboratory of Agricultural and Environmental Microbiology, Wuhan Institute of Virology, University of Chinese Academy of Sciences, Wuhan, Hubei, China.
3. Centre for Emerging Infectious Diseases, The State Key Laboratory of Virology, Wuhan Institute of Virology, University of Chinese Academy of Sciences, Wuhan, Hubei, China.

Corresponding authors:

Yiming SHAO, Division of Research on Virology and Immunology, National Center for AIDS/STD Control and Prevention, Chinese Center for Disease Control and Prevention, No. 155 Changbai Road, Changping District, Beijing 102206, China. Tel: +86 10 58900981; Fax: +86 10 58900980; E-mail: yshao08@gmail.com.

Zhiming YUAN, Key Laboratory of Agricultural and Environmental Microbiology, Wuhan Institute of Virology, University of Chinese Academy of Sciences, No. 44, Xiaohongshan, Wuhan, Hubei, 430071, China. Tel: +86 27 87197242; Fax: +86 27 87198120; E-mail: yzm@wh.iov.cn.

Condensed Title: Demographics of the current 2014 EBOV

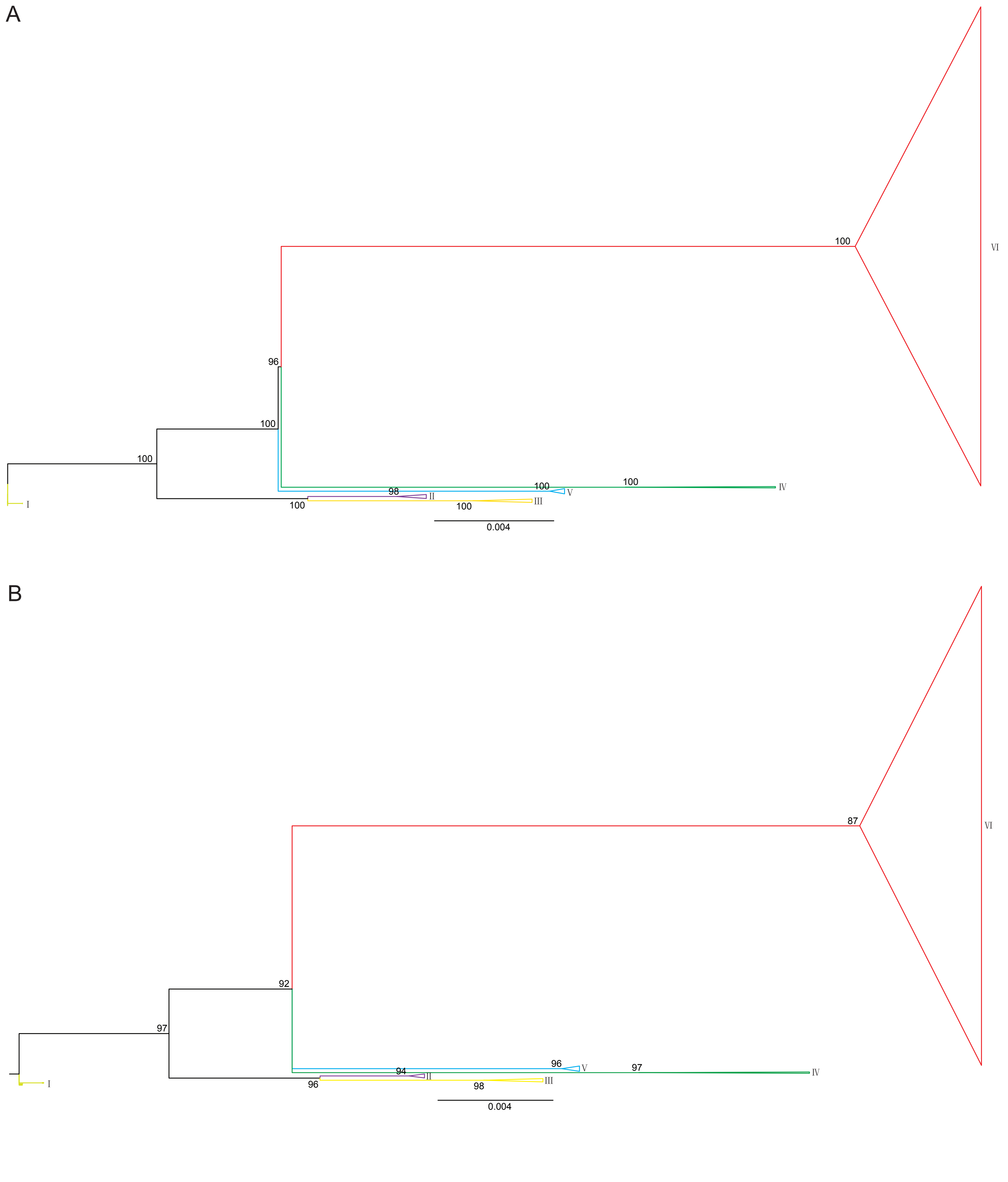

Supplementary Fig. S1. Phylogenetic relationship of EBOV between distinct outbreaks in West Africa. (A) Maximum likelihood (ML) phylogenetic tree of GP gene sequences of EBOV was constructed using RAxML. (B) Maximum likelihood (ML) phylogenetic tree of GP Gene sequences of EBOV was constructed using GARLI. All horizontal branch lengths are scaled to the number of nucleotide substitutions per site. Bootstrap values are shown for key nodes.

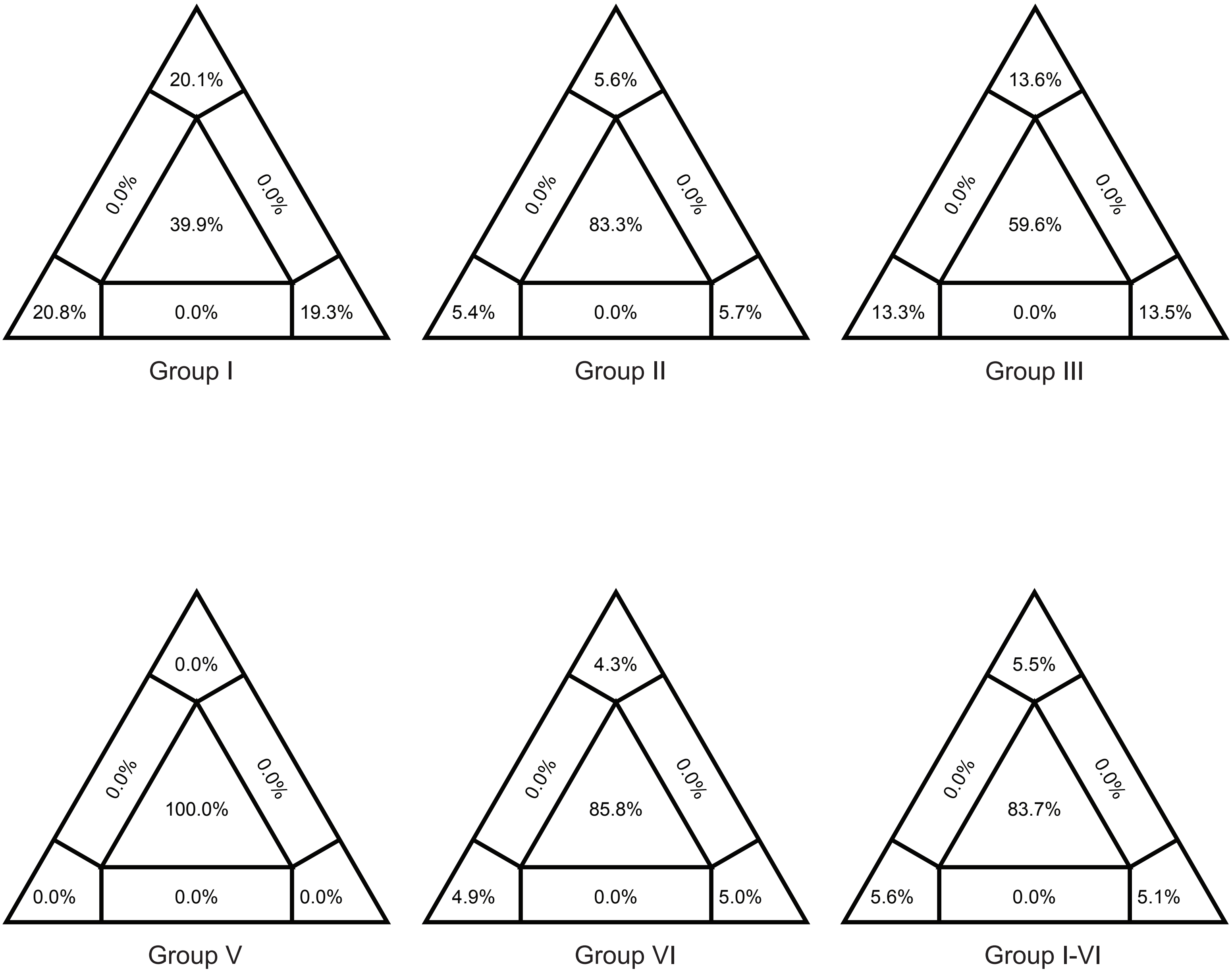

Supplementary Fig. S2. Likelihood-mapping analyses of EBOV epidemic waves. According to the lineages defined by the ML phylogenetic analyses of GP gene sequences of EBOV (Fig. S1), likelihood-mapping analyses were performed for each and overall lineages of EBOV epidemics with lineage IV excluded.

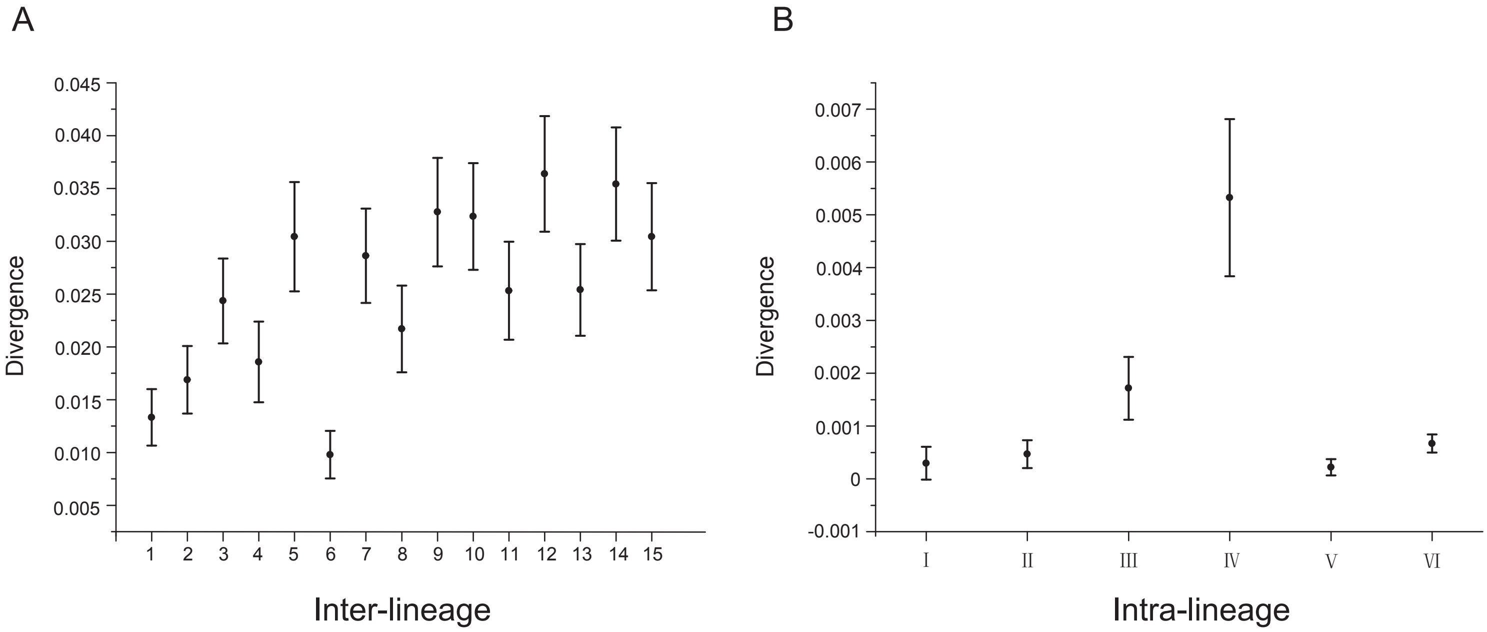

Supplementary Fig. S3. Comparison of inter-lineage and intra-lineage evolutionary divergence of GP gene sequences of EBOV. Inter-lineage (A) and intra-lineage (B) evolutionary divergence and standard error estimates of GP gene sequences of EBOV were calculated using maximum composite likelihood model with a gamma distribution.

Supplementary Table 1. List of Ebola virus glycoprotein (GP) sequences by country and collection used in the present study.			
**Accession number**	**Strain/Isolate name**	**Collection Country**	**Collection date**
KR063671	YambukuMayinga	Democratic Republic of Congo	1976-10-1
KR063672	Kikwit807223	Democratic Republic of Congo	1995-4-1
AY354458	Zaire	Democratic Republic of Congo	1995
HQ613402	034-KS	Democratic Republic of Congo	2008-12-31
HQ613403	M-M	Democratic Republic of Congo	2007-8-31
JQ352763	Kikwit	Democratic Republic of Congo	1995-5-4
KC242784	Luebo9	Democratic Republic of Congo	2007
KC242785	Lueb0	Democratic Republic of Congo	2007
KC242786	Luebo1	Democratic Republic of Congo	2007
KC242787	Luebo23	Democratic Republic of Congo	2007
KC242788	Luebo43	Democratic Republic of Congo	2007
KC242789	Luebo43	Democratic Republic of Congo	2007
KC242790	Luebo5	Democratic Republic of Congo	2007
KC242791	Bonduni	Democratic Republic of Congo	1977
KC242792	Gabon	Gabon	1994
KC242793	1Eko	Gabon	1996
KC242794	2Nza	Gabon	1996
KC242795	1Mbie	Gabon	1996
KC242796	13625Kikwit	Democratic Republic of Congo	1995
KC242797	1Oba	Gabon	1996
KC242798	1Ikot	Gabon	1996
KC242799	13709Kikwit	Democratic Republic of Congo	1995
KC242800	Ilembe	Gabon	2002
KC242801	deRoover	Democratic Republic of Congo	1976
KF113528	Kelle1	Democratic Republic of Congo	2003
KF113529	Kelle2	Democratic Republic of Congo	2003
KM655246	YambukuEcran	Democratic Republic of Congo	1976
KR824526	Zaire199510621	Democratic Republic of Congo	1995
KR867676	Kikwit9510621	Democratic Republic of Congo	1995-5-4
NC002549	YambukuMayinga	Democratic Republic of Congo	1976
KJ660346	C15	Guinea	2014-3-17
KJ660347	C07	Guinea	2014-3-20
KJ660348	C05	Guinea	2014-3-19
KP260799	DPR1	Mali	2014-10-23
KP260800	DPR2	Mali	2014-11-12
KP260801	DPR3	Mali	2014-11-21
KP260802	DPR4	Mali	2014-11-12
KP184503	UK1.1	Sierra Leone	2014-8-25
KP658432	UK2	Sierra Leone	2014-12-29
KP701371	INMI1	Sierra Leone	2014-11-25
KP728283	GE1	Sierra Leone	2014-11-21
KR025228	UK3	Sierra Leone	2015-3-12
KM034550	EM095	Sierra Leone	2014-5-25
KM034551	EM096	Sierra Leone	2014-5-26

KM034552	EM098	Sierra Leone	2014-5-26
KM034553	G3670.1	Sierra Leone	2014-5-27
KM034554	G3676.1	Sierra Leone	2014-5-27
KM034556	G3677.1	Sierra Leone	2014-5-26
KM034558	G3679.1	Sierra Leone	2014-5-28
KM034559	G3680.1	Sierra Leone	2014-5-28
KM034560	G3682.1	Sierra Leone	2014-5-28
KM034561	G3683.1	Sierra Leone	2014-5-28
KM034562	G3686.1	Sierra Leone	2014-5-28
KM034563	G3687.1	Sierra Leone	2014-5-28
KM233035	EM104	Sierra Leone	2014-6-2
KM233036	EM106	Sierra Leone	2014-6-2
KM233037	EM110	Sierra Leone	2014-6-3
KM233038	EM111	Sierra Leone	2014-6-3
KM233039	EM112	Sierra Leone	2014-6-3
KM233040	EM113	Sierra Leone	2014-6-3
KM233041	EM115	Sierra Leone	2014-6-3
KM233042	EM119	Sierra Leone	2014-6-3
KM233043	EM120	Sierra Leone	2014-6-3
KM233044	EM121	Sierra Leone	2014-6-4
KM233045	EM124.1	Sierra Leone	2014-6-4
KM233049	G3707	Sierra Leone	2014-5-31
KM233050	G3713.2	Sierra Leone	2014-6-9
KM233053	G3724	Sierra Leone	2014-6-5
KM233054	G3729	Sierra Leone	2014-6-7
KM233055	G3734.1	Sierra Leone	2014-6-7
KM233056	G3735.1	Sierra Leone	2014-6-7
KM233058	G3750.1	Sierra Leone	2014-6-10
KM233061	G3752	Sierra Leone	2014-6-10
KM233062	G3758	Sierra Leone	2014-6-11
KM233063	G3764	Sierra Leone	2014-6-12
KM233064	G3765.2	Sierra Leone	2014-6-14
KM233065	G3769.1	Sierra Leone	2014-6-12
KM233069	G3770.1	Sierra Leone	2014-6-12
KM233071	G3771	Sierra Leone	2014-6-12
KM233072	G3782	Sierra Leone	2014-6-14
KM233073	G3786	Sierra Leone	2014-6-14
KM233074	G3787	Sierra Leone	2014-6-14
KM233075	G3788	Sierra Leone	2014-6-14
KM233076	G3789.1	Sierra Leone	2014-6-14
KM233077	G3795	Sierra Leone	2014-6-15
KM233078	G3796	Sierra Leone	2014-6-15
KM233079	G3798	Sierra Leone	2014-6-15
KM233080	G3799	Sierra Leone	2014-6-15
KM233081	G3800	Sierra Leone	2014-6-15
KM233082	G3805.1	Sierra Leone	2014-6-15
KM233084	G3807	Sierra Leone	2014-6-15
KM233085	G3808	Sierra Leone	2014-6-15

KM233086	G3809	Sierra Leone	2014-6-15
KM233087	G3810.1	Sierra Leone	2014-6-15
KM233089	G3814	Sierra Leone	2014-6-15
KM233090	G3816	Sierra Leone	2014-6-15
KM233091	G3817	Sierra Leone	2014-6-15
KM233092	G3818	Sierra Leone	2014-6-15
KM233093	G3819	Sierra Leone	2014-6-15
KM233094	G3820	Sierra Leone	2014-6-15
KM233095	G3821	Sierra Leone	2014-6-15
KM233096	G3822	Sierra Leone	2014-6-15
KM233097	G3823	Sierra Leone	2014-6-15
KM233098	G3825.1	Sierra Leone	2014-6-16
KM233100	G3826	Sierra Leone	2014-6-16
KM233101	G3827	Sierra Leone	2014-6-16
KM233102	G3829	Sierra Leone	2014-6-16
KM233103	G3831	Sierra Leone	2014-6-16
KM233104	G3834	Sierra Leone	2014-6-17
KM233105	G3838	Sierra Leone	2014-6-17
KM233106	G3840	Sierra Leone	2014-6-17
KM233107	G3841	Sierra Leone	2014-6-17
KM233108	G3845	Sierra Leone	2014-6-18
KM233109	G3846	Sierra Leone	2014-6-18
KM233110	G3848	Sierra Leone	2014-6-18
KM233111	G3850	Sierra Leone	2014-6-18
KM233112	G3851	Sierra Leone	2014-6-18
KM233113	G3856.1	Sierra Leone	2014-6-18
KM233115	G3857	Sierra Leone	2014-6-18
KM233116	NM042.1	Sierra Leone	2014-6-4
KR534507	Conakry-505	Guinea	2014-7-24
KR534508	Conakry-509	Guinea	2014-7-24
KR534509	Siguiri-517	Guinea	2014-7-24
KR534510	Kouroussa-531	Guinea	2014-7-27
KR534511	Conakry-573	Guinea	2014-8-2
KR534512	Gueckedou-633	Guinea	2014-8-12
KR534513	Macenta-645	Guinea	2014-8-14
KR534514	Conakry-653	Guinea	2014-8-15
KR534515	Conakry-657	Guinea	2014-8-16
KR534516	Conakry-678	Guinea	2014-8-19
KR534517	Conakry-684	Guinea	2014-8-20
KR534518	Conakry-691	Guinea	2014-8-21
KR534519	Conakry-701	Guinea	2014-8-22
KR534520	Conakry-740	Guinea	2014-8-26
KR534521	Conakry-786	Guinea	2014-8-28
KR534522	Conakry-787	Guinea	2014-8-28
KR534523	Dubreka-789	Guinea	2014-8-29
KR534524	Coyah-955	Guinea	2014-9-11
KR534525	Conakry-976	Guinea	2014-9-14
KR534526	Forecariah-989	Guinea	2014-9-15

KR534527	Conakry-1043	Guinea	2014-9-19
KR534528	Kindia-1047	Guinea	2014-9-20
KR534529	Conakry-1059	Guinea	2014-9-21
KR534530	Coyah-1063	Guinea	2014-9-21
KR534531	Forecariah-1069	Guinea	2014-9-21
KR534532	Conakry-1081	Guinea	2014-9-22
KR534534	Dalaba-1116	Guinea	2014-9-24
KR534535	Conakry-1120	Guinea	2014-9-25
KR534536	Conakry-1121	Guinea	2014-9-25
KR534537	Conakry-1193	Guinea	2014-9-28
KR534538	Conakry-1205	Guinea	2014-10-2
KR534539	Conakry-1210	Guinea	2014-10-2
KR534540	Dalaba-1211	Guinea	2014-10-2
KR534541	Conakry-1249	Guinea	2014-10-4
KR534542	Coyah-1274	Guinea	2014-10-4
KR534543	Coyah-1277	Guinea	2014-10-4
KR534544	Coyah-1278	Guinea	2014-10-4
KR534545	Conakry-1298	Guinea	2014-10-6
KR534546	Coyah-1316	Guinea	2014-10-7
KR534548	Kerouane-1331	Guinea	2014-10-7
KR534549	Coyah-1333	Guinea	2014-10-7
KR534550	Coyah-1339	Guinea	2014-10-8
KR534551	Conakry-1340	Guinea	2014-10-8
KR534552	Conakry-1342	Guinea	2014-10-8
KR534553	Coyah-1355	Guinea	2014-10-9
KR534554	Forecariah-1365	Guinea	2014-10-9
KR534555	Conakry-1371	Guinea	2014-10-10
KR534556	Coyah-1374	Guinea	2014-10-10
KR534557	Coyah-1394	Guinea	2014-10-11
KR534558	Coyah-1436	Guinea	2014-10-13
KR534559	Conakry-1445	Guinea	2014-10-14
KR534560	Conakry-1454	Guinea	2014-10-14
KR534561	Conakry-1480	Guinea	2014-10-15
KR534562	Conakry-1551	Guinea	2014-10-18
KR534563	Conakry-1561	Guinea	2014-10-19
KR534564	Forecariah-1567	Guinea	2014-10-18
KR534565	Forecariah-1568	Guinea	2014-10-18
KR534566	Forecariah-1571	Guinea	2014-10-20
KR534567	Nzeerekore-1622	Guinea	2014-10-22
KR534568	Forecariah-1623	Guinea	2014-10-24
KR534569	Kindia-1648	Guinea	2014-10-23
KR534570	Conakry-1651	Guinea	2014-10-24
KR534571	Coyah-1652	Guinea	2014-10-24
KR534572	Coyah-1686	Guinea	2014-10-24
KR534573	Coyah-1689	Guinea	2014-10-25
KR534574	Coyah-1690	Guinea	2014-10-25
KR534575	Conakry-1481	Guinea	2014-10-15
KR534576	Conakry-1491	Guinea	2014-10-16

KR534577	Conakry-1027	Guinea	2014-9-18
KR534578	Conakry-1039	Guinea	2014-9-19
KR534579	Conakry-1105	Guinea	2014-9-24
KR534580	Conakry-1128	Guinea	2014-9-25
KR534581	Conakry-1129	Guinea	2014-9-25
KR534582	Conakry-1149	Guinea	2014-9-27
KR534583	Conakry-1213	Guinea	2014-10-2
KR534584	Conakry-1215	Guinea	2014-10-2
KR534585	Conakry-1250	Guinea	2014-10-4
KR534586	Conakry-507	Guinea	2014-7-24
KR534587	Conakry-742	Guinea	2014-8-26
KR534588	Conakry-768	Guinea	2014-8-27
KR534589	Coyah-1279	Guinea	2014-10-4
KR534590	Coyah-1320	Guinea	2014-10-7
KR534591	Coyah-1327	Guinea	2014-10-7
KP759594	J0109	Sierra Leone	2014-10-31
KP759595	J0111	Sierra Leone	2014-10-30
KP759596	J0114	Sierra Leone	2014-10-29
KP759597	J0115	Sierra Leone	2014-10-29
KP759598	J0118	Sierra Leone	2014-10-31
KP759599	J0120	Sierra Leone	2014-10-30
KP759600	J0129	Sierra Leone	2014-11-2
KP759601	J0132	Sierra Leone	2014-11-2
KP759602	J0135	Sierra Leone	2014-11-3
KP759603	J0140	Sierra Leone	2014-10-30
KP759604	J0143	Sierra Leone	2014-11-4
KP759605	J0144	Sierra Leone	2014-11-4
KP759606	J0030	Sierra Leone	2014-10-3
KP759607	J0031	Sierra Leone	2014-10-3
KP759608	J0034	Sierra Leone	2014-9-30
KP759609	J0035	Sierra Leone	2014-10-1
KP759610	J0146	Sierra Leone	2014-11-7
KP759611	J0147	Sierra Leone	2014-11-7
KP759612	J0148	Sierra Leone	2014-11-6
KP759613	J0151	Sierra Leone	2014-11-7
KP759614	J0152	Sierra Leone	2014-11-7
KP759615	J0037	Sierra Leone	2014-10-3
KP759616	J0153	Sierra Leone	2014-11-7
KP759617	J0154	Sierra Leone	2014-11-7
KP759618	J0038	Sierra Leone	2014-9-30
KP759619	J0159	Sierra Leone	2014-11-5
KP759620	J0039	Sierra Leone	2014-10-3
KP759621	J0160	Sierra Leone	2014-11-7
KP759622	J0162	Sierra Leone	2014-11-9
KP759623	J0164	Sierra Leone	2014-11-9
KP759624	J0167	Sierra Leone	2014-11-8
KP759625	J0170	Sierra Leone	2014-11-10
KP759626	J0172	Sierra Leone	2014-11-11

KP759627	J0173	Sierra Leone	2014-11-11
KP759628	J0005	Sierra Leone	2014-9-26
KP759629	J0040	Sierra Leone	2014-10-4
KP759630	J0006	Sierra Leone	2014-9-28
KP759631	J0007	Sierra Leone	2014-9-27
KP759632	J0047	Sierra Leone	2014-10-5
KP759633	J0051	Sierra Leone	2014-10-7
KP759634	J0056	Sierra Leone	2014-10-8
KP759635	J0057	Sierra Leone	2014-10-5
KP759636	J0001	Sierra Leone	2014-9-27
KP759637	J0061	Sierra Leone	2014-10-10
KP759638	J0063	Sierra Leone	2014-10-10
KP759639	J0010	Sierra Leone	2014-9-28
KP759640	J0002	Sierra Leone	2014-9-27
KP759641	J0011	Sierra Leone	2014-9-29
KP759642	J0012	Sierra Leone	2014-9-29
KP759643	J0013	Sierra Leone	2014-9-28
KP759644	J0018	Sierra Leone	2014-9-30
KP759645	J0085	Sierra Leone	2014-10-17
KP759646	J0086	Sierra Leone	2014-10-23
KP759647	J0087	Sierra Leone	2014-10-23
KP759648	J0088	Sierra Leone	2014-10-20
KP759649	J0090	Sierra Leone	2014-10-23
KP759650	J0092	Sierra Leone	2014-10-25
KP759651	J0003	Sierra Leone	2014-9-28
KP759652	J0023	Sierra Leone	2014-10-1
KP759653	J0095	Sierra Leone	2014-10-27
KP759654	J0096	Sierra Leone	2014-10-27
KP759655	J0100	Sierra Leone	2014-10-26
KP759656	J0101	Sierra Leone	2014-10-29
KP759657	J0102	Sierra Leone	2014-10-27
KP759658	J0103	Sierra Leone	2014-10-29
KP759659	J0108	Sierra Leone	2014-10-28
KP759660	J0110	Sierra Leone	2014-10-31
KP759661	J0112	Sierra Leone	2014-10-31
KP759662	J0113	Sierra Leone	2014-10-30
KP759663	J0024	Sierra Leone	2014-9-29
KP759664	J0116	Sierra Leone	2014-10-30
KP759665	J0117	Sierra Leone	2014-10-31
KP759666	J0025	Sierra Leone	2014-10-2
KP759667	J0119	Sierra Leone	2014-10-30
KP759668	J0004	Sierra Leone	2014-9-28
KP759669	J0121	Sierra Leone	2014-10-29
KP759670	J0026	Sierra Leone	2014-10-2
KP759671	J0122	Sierra Leone	2014-10-29
KP759672	J0123	Sierra Leone	2014-11-1
KP759673	J0124	Sierra Leone	2014-11-2
KP759674	J0125	Sierra Leone	2014-10-31

KP759675	J0126	Sierra Leone	2014-10-31
KP759676	J0127	Sierra Leone	2014-10-30
KP759677	J0128	Sierra Leone	2014-10-31
KP759678	J0027	Sierra Leone	2014-10-2
KP759679	J0130	Sierra Leone	2014-11-2
KP759680	J0131	Sierra Leone	2014-10-30
KP759681	J0133	Sierra Leone	2014-10-31
KP759682	J0134	Sierra Leone	2014-10-31
KP759683	J0028	Sierra Leone	2014-10-2
KP759684	J0136	Sierra Leone	2014-11-1
KP759685	J0137	Sierra Leone	2014-11-1
KP759686	J0138	Sierra Leone	2014-11-1
KP759687	J0139	Sierra Leone	2014-10-30
KP759688	J0029	Sierra Leone	2014-9-29
KP759689	J0141	Sierra Leone	2014-11-4
KP759690	J0142	Sierra Leone	2014-11-3
KP759691	J0032	Sierra Leone	2014-9-30
KP759692	J0033	Sierra Leone	2014-10-3
KP759693	J0145	Sierra Leone	2014-11-6
KP759694	J0036	Sierra Leone	2014-10-3
KP759695	J0149	Sierra Leone	2014-11-7
KP759696	J0150	Sierra Leone	2014-11-6
KP759697	J0155	Sierra Leone	2014-11-7
KP759698	J0156	Sierra Leone	2014-11-7
KP759699	J0157	Sierra Leone	2014-11-6
KP759700	J0158	Sierra Leone	2014-11-8
KP759701	J0161	Sierra Leone	2014-11-8
KP759702	J0163	Sierra Leone	2014-11-8
KP759703	J0165	Sierra Leone	2014-11-8
KP759704	J0166	Sierra Leone	2014-11-8
KP759705	J0168	Sierra Leone	2014-11-11
KP759706	J0169	Sierra Leone	2014-11-9
KP759707	J0171	Sierra Leone	2014-11-10
KP759708	J0174	Sierra Leone	2014-11-11
KP759709	J0175	Sierra Leone	2014-11-11
KP759710	J0041	Sierra Leone	2014-10-4
KP759711	J0042	Sierra Leone	2014-10-4
KP759712	J0043	Sierra Leone	2014-10-4
KP759713	J0044	Sierra Leone	2014-10-5
KP759714	J0045	Sierra Leone	2014-10-5
KP759715	J0046	Sierra Leone	2014-10-7
KP759716	J0048	Sierra Leone	2014-10-5
KP759717	J0049	Sierra Leone	2014-10-7
KP759718	J0008	Sierra Leone	2014-9-29
KP759719	J0050	Sierra Leone	2014-10-5
KP759720	J0052	Sierra Leone	2014-10-8
KP759721	J0053	Sierra Leone	2014-10-6
KP759722	J0054	Sierra Leone	2014-10-6

KP759723	J0055	Sierra Leone	2014-10-6
KP759724	J0058	Sierra Leone	2014-10-8
KP759725	J0059	Sierra Leone	2014-10-8
KP759726	J0060	Sierra Leone	2014-10-6
KP759727	J0062	Sierra Leone	2014-10-10
KP759728	J0064	Sierra Leone	2014-10-9
KP759729	J0065	Sierra Leone	2014-10-9
KP759730	J0066	Sierra Leone	2014-10-10
KP759731	J0067	Sierra Leone	2014-10-9
KP759732	J0068	Sierra Leone	2014-10-9
KP759733	J0069	Sierra Leone	2014-10-9
KP759734	J0009	Sierra Leone	2014-9-28
KP759735	J0070	Sierra Leone	2014-10-9
KP759736	J0071	Sierra Leone	2014-10-9
KP759737	J0072	Sierra Leone	2014-10-9
KP759738	J0073	Sierra Leone	2014-10-12
KP759739	J0074	Sierra Leone	2014-10-13
KP759740	J0014	Sierra Leone	2014-9-29
KP759741	J0015	Sierra Leone	2014-9-26
KP759742	J0016	Sierra Leone	2014-9-30
KP759743	J0075	Sierra Leone	2014-10-17
KP759744	J0076	Sierra Leone	2014-10-17
KP759745	J0077	Sierra Leone	2014-10-17
KP759746	J0078	Sierra Leone	2014-10-18
KP759747	J0017	Sierra Leone	2014-9-26
KP759748	J0079	Sierra Leone	2014-10-16
KP759749	J0080	Sierra Leone	2014-10-18
KP759750	J0081	Sierra Leone	2014-10-16
KP759751	J0082	Sierra Leone	2014-10-16
KP759752	J0083	Sierra Leone	2014-10-18
KP759753	J0084	Sierra Leone	2014-10-16
KP759754	J0019	Sierra Leone	2014-9-25
KP759755	J0020	Sierra Leone	2014-9-25
KP759756	J0021	Sierra Leone	2014-9-29
KP759757	J0022	Sierra Leone	2014-9-25
KP759758	J0089	Sierra Leone	2014-10-23
KP759759	J0091	Sierra Leone	2014-10-25
KP759760	J0093	Sierra Leone	2014-10-24
KP759761	J0094	Sierra Leone	2014-10-27
KP759762	J0097	Sierra Leone	2014-10-28
KP759763	J0098	Sierra Leone	2014-10-28
KP759764	J0099	Sierra Leone	2014-10-28
KP759765	J0104	Sierra Leone	2014-10-29
KP759766	J0105	Sierra Leone	2014-10-29
KP759767	J0106	Sierra Leone	2014-10-28
KP759768	J0107	Sierra Leone	2014-10-29
KR817067	EM000015	Guinea	2014-9-1
KR817068	EM000027	Guinea	2014-9-1

KR817069	EM000028	Guinea	2014-9-1
KR817070	EM000127	Guinea	2014-9-4
KR817071	EM000128	Guinea	2014-9-4
KR817072	EM000218	Guinea	2014-9-7
KR817073	EM000219	Guinea	2014-9-7
KR817074	EM000321	Guinea	2014-9-9
KR817075	EM000457	Guinea	2014-9-12
KR817076	EM000500	Guinea	2014-9-13
KR817077	EM000501	Guinea	2014-9-13
KR817078	EM000502	Guinea	2014-9-13
KR817079	EM000706	Guinea	2014-9-21
KR817080	EM000707	Guinea	2014-9-22
KR817081	EM000921	Guinea	2014-9-29
KR817082	EM000925	Guinea	2014-9-29
KR817083	EM000934	Guinea	2014-9-29
KR817084	EM000958	Guinea	2014-9-30
KR817085	EM000968	Guinea	2014-10-1
KR817086	EM000982	Guinea	2014-10-1
KR817087	EM000983	Guinea	2014-10-1
KR817088	EM001101	Guinea	2014-10-5
KR817089	EM001102	Guinea	2014-10-5
KR817090	EM004059	Guinea	2014-12-20
KR817091	EM004085	Guinea	2014-12-19
KR817092	EM004192	Guinea	2014-12-22
KR817093	EM004201	Guinea	2014-12-24
KR817094	EM004259	Guinea	2014-12-26
KR817095	EM004290	Guinea	2014-12-27
KR817096	EM004414	Guinea	2015-1-2
KR817097	EM004422	Guinea	2015-1-2
KR817098	EM004437	Guinea	2015-1-4
KR817099	EM004438	Guinea	2015-1-4
KR817100	EM004481	Guinea	2015-1-11
KR817101	EM004494	Guinea	2015-1-14
KR817102	EM004503	Guinea	2015-1-15
KR817103	EM004555	Guinea	2015-1-22
KR817104	EM004563	Guinea	2015-1-25
KR817105	EM004580	Guinea	2015-1-27
KR817106	EM004589	Guinea	2015-1-31
KR817107	EM074335	Guinea	2014-7-18
KR817108	EM074349	Liberia	2014-7-22
KR817109	EM074350	Liberia	2014-7-22
KR817110	EM074351	Liberia	2014-7-22
KR817111	EM074352	Liberia	2014-7-22
KR817112	EM074353	Liberia	2014-7-22
KR817113	EM074354	Guinea	2014-7-22
KR817114	EM074391	Liberia	2014-7-26
KR817115	EM074392	Liberia	2014-7-25
KR817116	EM074436	Guinea	2014-8-1

KR817117	EM074437	Guinea	2014-8-1
KR817118	EM074438	Guinea	2014-8-1
KR817119	EM074439	Guinea	2014-8-1
KR817120	EM074461	Guinea	2014-8-4
KR817121	EM074462	Guinea	2014-8-4
KR817122	EM074531	Guinea	2014-8-8
KR817123	EM074548	Liberia	2014-8-8
KR817124	EM074684	Guinea	2014-8-14
KR817125	EM074720	Liberia	2014-8-14
KR817126	EM074785	Guinea	2014-8-16
KR817127	EM074821	Liberia	2014-8-17
KR817128	EM074822	Liberia	2014-8-17
KR817129	EM075043	Liberia	2014-8-22
KR817130	EM075076	Guinea	2014-8-23
KR817131	EM075368	Guinea	2014-8-29
KR817132	EM075373	Guinea	2014-8-30
KR817133	EM075435	Guinea	2014-8-30
KR817134	EM075447	Guinea	2014-8-31
KR817135	EM075928	Guinea	2014-10-10
KR817136	EM075929	Guinea	2014-10-10
KR817137	EM075930	Guinea	2014-10-10
KR817138	EM075931	Guinea	2014-10-10
KR817139	EM075932	Guinea	2014-10-10
KR817140	EM076138	Guinea	2014-10-17
KR817141	EM076191	Guinea	2014-10-18
KR817142	EM076192	Guinea	2014-10-18
KR817143	EM076193	Guinea	2014-10-18
KR817144	EM076217	Guinea	2014-10-19
KR817145	EM076322	Guinea	2014-10-22
KR817146	EM076334	Guinea	2014-10-23
KR817147	EM076335	Guinea	2014-10-23
KR817148	EM076383	Guinea	2014-10-26
KR817149	EM076403	Guinea	2014-10-27
KR817150	EM076472	Guinea	2014-10-29
KR817151	EM076533	Guinea	2014-11-1
KR817152	EM076534	Guinea	2014-11-1
KR817153	EM076610	Guinea	2014-11-3
KR817155	EM076769	Guinea	2014-11-8
KR817156	EM076770	Guinea	2014-11-8
KR817157	EM076948	Guinea	2014-11-13
KR817158	EM076949	Guinea	2014-11-13
KR817159	EM076951	Guinea	2014-11-13
KR817160	EM078415	Guinea	2014-11-24
KR817161	EM078416	Guinea	2014-11-24
KR817162	EM078555	Guinea	2014-12-1
KR817163	EM078556	Guinea	2014-12-1
KR817164	EM078608	Guinea	2014-12-4
KR817165	EM078638	Guinea	2014-12-3

KR817166	EM078639	Guinea	2014-12-4
KR817167	EM078654	Guinea	2014-12-9
KR817168	EM078656	Guinea	2014-12-9
KR817169	EM078670	Guinea	2014-12-11
KR817170	EM078683	Guinea	2014-12-11
KR817171	EM078694	Guinea	2014-12-14
KR817172	EM078697	Guinea	2014-12-14
KR817173	EM078706	Guinea	2014-12-15
KR817174	EM078709	Guinea	2014-12-14
KR817175	EM078722	Guinea	2014-12-16
KR817176	EM078763	Guinea	2014-12-17
KR817177	EM078779	Guinea	2014-12-18
KR817179	EM079404	Guinea	2014-3-28
KR817180	EM079405	Guinea	2014-3-28
KR817181	EM079408	Guinea	2014-3-31
KR817182	EM079410	Guinea	2014-3-31
KR817183	EM079412	Guinea	2014-3-31
KR817184	EM079413	Guinea	2014-3-31
KR817185	EM079414	Guinea	2014-3-31
KR817186	EM079421	Guinea	2014-3-30
KR817187	EM079422	Guinea	2014-3-27
KR817188	EM079423	Guinea	2014-3-27
KR817189	EM079424	Guinea	2014-3-27
KR817190	EM079429	Guinea	2014-4-2
KR817191	EM079434	Guinea	2014-4-2
KR817192	EM079442	Guinea	2014-4-3
KR817193	EM079444	Guinea	2014-4-2
KR817194	EM079450	Liberia	2014-4-1
KR817195	EM079464	Guinea	2014-4-4
KR817196	EM079497	Guinea	2014-4-7
KR817197	EM079514	Guinea	2014-4-10
KR817198	EM079517	Guinea	2014-4-11
KR817199	EM079542	Guinea	2014-4-12
KR817200	EM079549	Guinea	2014-4-13
KR817201	EM079578	Guinea	2014-4-18
KR817202	EM079587	Guinea	2014-4-22
KR817203	EM079622	Guinea	2014-4-28
KR817204	EM079630	Guinea	2014-5-1
KR817205	EM079657	Guinea	2014-5-7
KR817206	EM079659	Guinea	2014-5-7
KR817207	EM079677	Guinea	2014-5-10
KR817208	EM079681	Guinea	2014-5-11
KR817209	EM079685	Guinea	2014-5-11
KR817210	EM079702	Guinea	2014-5-14
KR817211	EM079731	Guinea	2014-5-18
KR817212	EM079749	Guinea	2014-5-21
KR817213	EM079750	Guinea	2014-5-21
KR817214	EM079753	Guinea	2014-5-22

KR817215	EM079772	Guinea	2014-5-24
KR817216	EM079775	Guinea	2014-5-24
KR817217	EM079815	Guinea	2014-5-28
KR817218	EM079859	Guinea	2014-6-1
KR817219	EM079876	Guinea	2014-6-5
KR817220	EM079880	Guinea	2014-6-5
KR817221	EM079910	Guinea	2014-6-9
KR817222	EM079911	Guinea	2014-6-9
KR817223	EM079912	Guinea	2014-6-9
KR817224	EM079913	Guinea	2014-6-9
KR817225	EM079914	Guinea	2014-6-10
KR817226	EM079915	Guinea	2014-6-10
KR817227	EM079983	Sierra Leone	2014-6-13
KR817228	EM080003	Sierra Leone	2014-6-14
KR817229	EM080011	Sierra Leone	2014-6-15
KR817230	EM080063	Guinea	2014-6-20
KR817231	EM080064	Liberia	2014-6-20
KR817232	EM080065	Liberia	2014-6-20
KR817233	EM080066	Liberia	2014-6-20
KR817234	EM080067	Liberia	2014-6-20
KR817235	EM080076	Guinea	2014-6-22
KR817236	EM080132	Sierra Leone	2014-6-24
KR817237	EM080141	Guinea	2014-6-25
KR817238	EM080165	Sierra Leone	2014-6-26
KR817239	EM080193	Liberia	2014-6-29
KR817240	EM080213	Liberia	2014-7-3
KR817242	EM080253	Guinea	2014-7-10
KR817243	EM080261	Liberia	2014-7-12
KR817244	EM080265	Sierra Leone	2014-7-12
KR817245	EM080269	Liberia	2014-7-12
KR105200	G3838.2	Sierra Leone	2014-6-16
KR105202	G3851.2	Sierra Leone	2014-6-18
KR105204	G3886.1	Sierra Leone	2014-6-19
KR105205	G3889.1	Sierra Leone	2014-6-19
KR105206	G3913.1	Sierra Leone	2014-6-21
KR105207	G3917.1	Sierra Leone	2014-6-21
KR105208	G3926.2	Sierra Leone	2014-6-22
KR105209	G3949.1	Sierra Leone	2014-6-24
KR105210	G3950.1	Sierra Leone	2014-6-24
KR105213	G4132.1	Sierra Leone	2014-7-5
KR105214	G4133.1	Sierra Leone	2014-7-4
KR105215	G4151.1	Sierra Leone	2014-7-6
KR105216	G4190.1	Sierra Leone	2014-7-7
KR105217	G4200.1	Sierra Leone	2014-7-8
KR105218	G4217.1	Sierra Leone	2014-7-8
KR105219	G4221.1	Sierra Leone	2014-7-9
KR105221	G4236.1	Sierra Leone	2014-7-10
KR105222	G4250.1	Sierra Leone	2014-7-11

KR105223	G4251.1	Sierra Leone	2014-7-11
KR105224	G4252.1	Sierra Leone	2014-7-11
KR105225	G4254.1	Sierra Leone	2014-7-11
KR105226	G4255.1	Sierra Leone	2014-7-11
KR105227	G4263.1	Sierra Leone	2014-7-11
KR105228	G4264.1	Sierra Leone	2014-7-11
KR105229	G4299.1	Sierra Leone	2014-7-12
KR105230	G4312.2	Sierra Leone	2014-7-12
KR105231	G4316.1	Sierra Leone	2014-7-13
KR105232	G4323.2	Sierra Leone	2014-7-14
KR105233	G4324.1	Sierra Leone	2014-7-14
KR105234	G4325.1	Sierra Leone	2014-7-14
KR105235	G4329.1	Sierra Leone	2014-7-14
KR105236	G4333.1	Sierra Leone	2014-7-13
KR105237	G4334.1	Sierra Leone	2014-7-14
KR105238	G4337.1	Sierra Leone	2014-7-14
KR105239	G4345.1	Sierra Leone	2014-7-18
KR105240	G4347.1	Sierra Leone	2014-7-15
KR105241	G4348.1	Sierra Leone	2014-7-9
KR105242	G4350.1	Sierra Leone	2014-7-14
KR105243	G4380.1	Sierra Leone	2014-7-15
KR105244	G4382.1	Sierra Leone	2014-7-16
KR105247	G4415.1	Sierra Leone	2014-7-18
KR105248	G4416.1	Sierra Leone	2014-7-18
KR105249	G4419.1	Sierra Leone	2014-7-19
KR105250	G4422.1	Sierra Leone	2014-7-19
KR105251	G4423.1	Sierra Leone	2014-7-19
KR105252	G4424.1	Sierra Leone	2014-7-19
KR105256	G4445.1	Sierra Leone	2014-7-21
KR105258	G4454.1	Sierra Leone	2014-7-21
KR105262	G4683.1	Sierra Leone	2014-8-1
KR105263	G4698.1	Sierra Leone	2014-8-3
KR105264	G4701.1	Sierra Leone	2014-8-3
KR105265	G4702.1	Sierra Leone	2014-8-3
KR105266	G4717.1	Sierra Leone	2014-8-4
KR105268	G4730.1	Sierra Leone	2014-8-5
KR105269	G4736.1	Sierra Leone	2014-8-5
KR105270	G4748.1	Sierra Leone	2014-8-5
KR105271	G4751.1	Sierra Leone	2014-8-5
KR105273	G4856.1	Sierra Leone	2014-8-10
KR105274	G4861.1	Sierra Leone	2014-8-10
KR105275	G4868.1	Sierra Leone	2014-8-10
KR105276	G4886.1	Sierra Leone	2014-8-11
KR105277	G4907.1	Sierra Leone	2014-8-12
KR105278	G4937.1	Sierra Leone	2014-8-13
KR105279	G4942.1	Sierra Leone	2014-8-12
KR105280	G4946.1	Sierra Leone	2014-8-13
KR105281	G4955.1	Sierra Leone	2014-8-13

KR105283	G4960.1	Sierra Leone	2014-8-14
KR105284	G4971.1	Sierra Leone	2014-8-14
KR105285	G4972.1	Sierra Leone	2014-8-14
KR105286	G4973.1	Sierra Leone	2014-8-12
KR105287	G4981.1	Sierra Leone	2014-8-14
KR105288	G4982.1	Sierra Leone	2014-8-14
KR105289	G4994.1	Sierra Leone	2014-8-15
KR105290	G4996.1	Sierra Leone	2014-8-15
KR105291	G4999.1	Sierra Leone	2014-8-15
KR105292	G5012.3	Sierra Leone	2014-8-15
KR105293	G5016.1	Sierra Leone	2014-8-16
KR105294	G5019.1	Sierra Leone	2014-8-16
KR105295	G5039.1	Sierra Leone	2014-8-17
KR105298	G5112.1	Sierra Leone	2014-8-19
KR105300	G5119.1	Sierra Leone	2014-8-24
KR105301	G5244.1	Sierra Leone	2014-8-22
KR105303	G5296.1	Sierra Leone	2014-8-25
KR105306	G5370.1	Sierra Leone	2014-8-28
KR105307	G5516.1	Sierra Leone	2014-9-4
KR105308	G5520.1	Sierra Leone	2014-9-5
KR105310	G5570.1	Sierra Leone	2014-9-8
KR105311	G5571.1	Sierra Leone	2014-9-8
KR105312	G5617.1	Sierra Leone	2014-9-10
KR105313	G5640.1	Sierra Leone	2014-9-11
KR105315	G5647.1	Sierra Leone	2014-9-11
KR105316	G5684.1	Sierra Leone	2014-9-13
KR105317	G5685.1	Sierra Leone	2014-9-13
KR105318	G5691.1	Sierra Leone	2014-9-14
KR105320	G5731.1	Sierra Leone	2014-9-16
KR105321	G5737.1	Sierra Leone	2014-9-16
KR105322	G5738.1	Sierra Leone	2014-9-15
KR105323	G5743.1	Sierra Leone	2014-9-17
KR105324	G5756.1	Sierra Leone	2014-9-18
KR105325	G5763.1	Sierra Leone	2014-9-16
KR105326	G5765.1	Sierra Leone	2014-9-16
KR105327	G5767.1	Sierra Leone	2014-9-16
KR105328	G5844.1	Sierra Leone	2014-9-21
KR105329	G5853.1	Sierra Leone	2014-9-21
KR105330	G5879.1	Sierra Leone	2014-9-22
KR105331	G5898.1	Sierra Leone	2014-9-22
KR105332	G5982.1	Sierra Leone	2014-9-25
KR105333	G5983.1	Sierra Leone	2014-9-25
KR105335	G5986.1	Sierra Leone	2014-9-25
KR105337	G5996.1	Sierra Leone	2014-9-25
KR105338	G5997.1	Sierra Leone	2014-9-25
KR105339	G5998.1	Sierra Leone	2014-9-25
KR105340	G6012.1	Sierra Leone	2014-9-25
KR105341	G6020.1	Sierra Leone	2014-9-25

KR105342	G6060.1	Sierra Leone	2014-9-25
KR105343	G6062.1	Sierra Leone	2014-9-25
KR105344	G6069.1	Sierra Leone	2014-9-25
KR105345	G6089.1	Sierra Leone	2014-9-27
KR105346	G6091.1	Sierra Leone	2014-9-27
KR105347	G6095.1	Sierra Leone	2014-9-27
KR105349	G6104.1	Sierra Leone	2014-9-28
KR653224	20146553	Sierra Leone	2014-12-22
KR653225	20141650	Sierra Leone	2014-10-5
KR653226	20142551	Sierra Leone	2014-10-18
KR653227	20140134	Sierra Leone	2014-8-26
KR653228	20143550	Sierra Leone	2014-11-1
KR653229	20141282	Sierra Leone	2014-9-23
KR653230	20141582	Sierra Leone	2014-10-3
KR653231	20144865	Sierra Leone	2014-11-13
KR653232	20141288	Sierra Leone	2014-9-23
KR653233	20141643	Sierra Leone	2014-10-4
KR653234	20142127	Sierra Leone	2014-10-12
KR653235	20140489	Sierra Leone	2014-9-4
KR653236	20143753	Sierra Leone	2014-11-5
KR653237	20146001	Sierra Leone	2014-11-24
KR653238	20144192	Sierra Leone	2014-11-8
KR653239	20140091	Sierra Leone	2014-8-22
KR653240	20143918	Sierra Leone	2014-11-6
KR653241	20140100	Sierra Leone	2014-8-24
KR653242	20143964	Sierra Leone	2014-11-7
KR653243	20144820	Sierra Leone	2014-11-15
KR653244	20141012	Sierra Leone	2014-9-21
KR653245	20143458	Sierra Leone	2014-11-1
KR653246	20140433	Sierra Leone	2014-9-3
KR653247	20143107	Sierra Leone	2014-10-25
KR653248	20143415	Sierra Leone	2014-10-31
KR653249	20142407	Sierra Leone	2014-10-16
KR653250	20141061	Sierra Leone	2014-9-21
KR653251	20140008	Sierra Leone	2014-8-22
KR653252	20140024	Sierra Leone	2014-8-20
KR653253	20143648	Sierra Leone	2014-11-3
KR653254	20142417	Sierra Leone	2014-10-18
KR653255	20142477	Sierra Leone	2014-10-19
KR653256	20143659	Sierra Leone	2014-11-3
KR653257	20143796	Sierra Leone	2014-11-5
KR653258	20143360	Sierra Leone	2014-10-30
KR653259	20144837	Sierra Leone	2014-11-14
KR653260	20141429	Sierra Leone	2014-9-28
KR653261	20141271	Sierra Leone	2014-9-24
KR653262	20142260	Sierra Leone	2014-10-14
KR653263	20140517	Sierra Leone	2014-9-5
KR653264	20143938	Sierra Leone	2014-11-7

KR653265	20140161	Sierra Leone	2014-8-27
KR653266	20141232	Sierra Leone	2014-9-25
KR653267	20140038	Sierra Leone	2014-8-23
KR653268	20142895	Sierra Leone	2014-10-24
KR653269	20140910	Sierra Leone	2014-9-18
KR653270	20144610	Sierra Leone	2014-11-12
KR653271	20144521	Sierra Leone	2014-11-12
KR653272	20143018	Sierra Leone	2014-10-26
KR653273	20141497	Sierra Leone	2014-10-1
KR653274	20142065	Sierra Leone	2014-10-11
KR653275	20143031	Sierra Leone	2014-10-25
KR653276	20141280	Sierra Leone	2014-9-23
KR653277	20143164	Sierra Leone	2014-10-27
KR653278	20140590	Sierra Leone	2014-9-7
KR653279	20140395	Sierra Leone	2014-9-2
KR653280	20140933	Sierra Leone	2014-9-16
KR653282	20143317	Sierra Leone	2014-10-29
KR653283	20141960	Sierra Leone	2014-10-9
KR653284	20141352	Sierra Leone	2014-9-26
KR653285	20143036	Sierra Leone	2014-10-24
KR653286	20140729	Sierra Leone	2014-9-10
KR653287	20140436	Sierra Leone	2014-9-3
KR653288	20141397	Sierra Leone	2014-9-28
KR653289	20141491	Sierra Leone	2014-10-1
KR653290	20145853	Sierra Leone	2014-11-26
KR653291	20141123	Sierra Leone	2014-9-22
KR653292	20143716	Sierra Leone	2014-11-4
KR653293	20141997	Sierra Leone	2014-10-10
KR653294	20140174	Sierra Leone	2014-8-27
KR653295	20145835	Sierra Leone	2014-11-25
KR653296	20140254	Sierra Leone	2014-8-29
KR653297	20140872	Sierra Leone	2014-9-15
KR653298	20143187	Sierra Leone	2014-10-28
KR653299	20144819	Sierra Leone	2014-11-15
KR653300	20141241	Sierra Leone	2014-9-24
KR653301	20142843	Sierra Leone	2014-10-23
KR653302	20142856	Sierra Leone	2014-10-23
KR653303	20141227	Sierra Leone	2014-9-26
KR653304	20143466	Sierra Leone	2014-11-1
KR653305	20141043	Sierra Leone	2014-9-21

Supplementary Table 2. Detecting selection pressures by codon substitution models for GP gene of EBOV.

Gene	Class of models	Model	lnL	Compared models	df	LRT	*p*-value	Positive selected sites
		Two-ratio Model 2	-4680.75569					
	Branch-specific models	One-ratio Model 0	-4683.32627	Two-ratio Model 2 - One-ratio Model	1	5.141164	0.023365*	NA
		M3	-4668.13079					
		M0	-4683.32627	M3 - M0	4	30.390962	0.000005*	NA
		M2a	-4668.14766					
		M1a	-4670.42052	M2a - M1a	2	4.54573	0.103017	-
		M8	-4668.138					
		M7	-4670.9349	M8 - M7	2	5.593808	0.060999	-
		M8	-4668.138					
	Site-specific models	M8a	-4670.45714	M8 - M8a	1	4.63829	0.031267*	NA
		Model A	-4670.42057					
		M1a	-4670.42052	Model A - M1a	2	0	1	NA
		Model A	-4670.42057					
		Model A1	-4670.42054	Model A - Model A1	1	0	1	-
		Model A1	-4670.42054					
		M1a	-4670.42052	Model A1 - M1a	1	0	1	NA
		Model D (*k* =3)	-4665.12621					
GP	Branch-site models	M3	-4668.13079	Model D - M3	1	6.009164	0.014232*	NA

*Indicates the difference is significant at $p < 0.05$.
